# Light Regulation of Carotenoid Biosynthesis in the Peel of Mandarin and Sweet Orange Fruits

**DOI:** 10.3389/fpls.2019.01288

**Published:** 2019-10-15

**Authors:** Joanna Lado, Enriqueta Alós, Matías Manzi, Paul J.R. Cronje, Aurelio Gómez-Cadenas, María J. Rodrigo, Lorenzo Zacarías

**Affiliations:** ^1^Instituto de Agroquímica y Tecnología de Alimentos (IATA), Consejo Superior de Investigaciones Científicas (CSIC), Valencia, Spain; ^2^Instituto Nacional de Investigación Agropecuaria (INIA), Camino a la Represa s/n, Salto, Uruguay; ^3^Departament de Ciències Agràries i del Medi Natural, Universitat Jaume I, Castelló de la Plana, Spain; ^4^Estación Experimental Facultad de Agronomía Salto (EEFAS), Universidad de la Republica, Salto, Uruguay; ^5^Citrus Research International, Department of Horticultural Science, University of Stellenbosch, Stellenbosch, South Africa

**Keywords:** abscisic acid, carotenoids, citrus fruits, gene expression, light, maturation

## Abstract

Carotenoids are the pigments responsible for the coloration of the peel and pulp of *Citrus* fruits. Light is one of the major environmental factors influencing coloration and carotenoid content and composition of fleshy fruits and therefore their commercial and nutritional quality. Agronomical observations indicate that citrus fruits exposed to sunlight develop a brighter peel coloration than shaded fruit inside the tree canopy. In the present study, the effect of light deprivation on carotenoid profile, and in the expression of genes of carotenoid metabolism and their precursors have been analyzed in fruits of Clemenules mandarin (*Citrus clementine*) and Navelina orange (*Citrus sinensis*). Fruit shading accelerated peel degreening, chlorophyll degradation, and reduced chloroplastic-type carotenoids. Time-course shading experiments revealed that the stage of fruit ripening appears to be determinant for the effect of darkness in carotenoid biosynthesis. Fruit shading produced a down-regulation of the expression of key carotenoids biosynthetic genes (*PSY*, *PDS*, *ZDS1*, *LCY2a*, *LCY2b*, and *CHX*). However, expression of MEP pathway genes (*DXS*, *HDR1*, and *GGPPS1*) and the carotenoid cleavage dioxygenase, *CCD4b1*, responsible of the formation of the apocarotenoid β-citraurin, were not substantially affected by dark-grown conditions. The content of abscisic acid (ABA), an end product of the carotenoid pathway, was not affected by the light regime, suggesting that effect of shading on the precursor’s pool is not sufficient to impair ABA synthesis. A moderate increase in total carotenoid and in the expression of biosynthetic genes was observed in mature dark-grown mandarin and orange fruits. Collectively, results suggest that light stimulates carotenoid biosynthesis in the peel of citrus fruits but a light-independent regulation may also operate.

## Introduction

Fruit color is a crucial parameter for quality standards, marketability, and consumer acceptance, and of particular relevance for fruit destined to fresh consumption (Lado et al., 2018). The color of Citrus fruits is highly diverse and mainly determined by differences in carotenoid content and composition (Rodrigo et al., 2013a; Ikoma et al., 2016; Wei et al., 2017). Carotenoids are the main pigments accumulated in the peel and pulp of citrus fruit and beside providing coloration they also exert different functions related to energy capture and dissipation during photosynthesis, are precursors of the plant hormones ABA and strigolactones, among others, and render nutritional and health-related properties by their antioxidant and pro-vitamin A capacity (Rodríguez-Concepción et al., 2018).

The regulation of carotenoid biosynthesis in *Citrus* fruits has been exhaustively investigated during the last decade but how genetic and environmental cues determinate the natural diversity in fruit coloration and carotenoids composition are not fully understood (Alquézar et al., 2008; Kato, 2012; Rodrigo et al., 2013a; Ikoma et al., 2016). The flavedo of immature citrus fruit has a composition that resembles green vegetative tissues and accumulates mainly lutein and minor amounts of other chloroplastic carotenoids (zeaxanthin, neoxanthin, β- and α-carotene). During natural ripening, lutein declines or almost disappears in parallel to a marked increment of colored carotenoids, mostly β,β-xanthophylls (violxanthin isomers and β-cryptoxanthin), which are the predominant compounds found in orange-colored mature citrus fruit (Alquézar et al., 2008; Kato, 2012; Rodrigo et al., 2013a). Interestingly, the peel of sweet oranges and mandarins accumulates during ripening citrus-specific C_30_ apocarotenoids, mainly β-citraurin, derived from the enzymatic cleavage of zeaxanthin and β-cryptoxanthin, and their contents are highly correlated with the orange-reddish pigmentation of the peel (Ma et al., 2013; Rodrigo et al., 2013b). All these changes in carotenoids composition occur alongside the induction of the expression of carotenoid biosynthetic genes: first there is an increase in the transcript levels of phytoene synthase (*PSY*), the first rate limiting and committed step of carotenoid biosynthesis, followed by the stimulation of phytoene desaturase (*PDS*), ζ-carotene desaturase (*ZDS*), β-carotene hydroxylase (β-CHX) and the chromoplast-specific lycopene cyclase 2 (βLCY2) (Rodrigo et al., 2013a; Ikoma et al., 2016; Wei et al., 2017). The biosynthesis of the C_30_ apocarotenoid β-citraurin is mediated by the action of a specific isoform of the carotenoid cleavage dioxygenase type 4 (*CCD4b1*) whose expression is restricted to the peel of citrus fruit and induced during ripening (Ma et al., 2013; Rodrigo et al., 2013b). The metabolic precursors of carotenoids are synthetized by the methyl-D-erythritol-4-phosphate (MEP) pathway. The flux into the MEP pathway is mainly controlled by two key enzymes: the 1-deoxy-D-xylulose-5-phosphate synthase (DXS), located upstream, and the hydroxymethylbutenyl diphosphate reductase (HDR), downstream in the pathway, constituting two regulatory steps that directly influence carotenoid accumulation (Rodríguez-Concepción et al., 2018). At the end of the MEP pathway, the formation of the key precursor geranyl geranyl pyrophosphate (GGPP), which constitutes a key branching point in the biosynthesis of isoprenoids in plants, is catalyzed by GGPP synthases (GGPPs), a complex family of different isoforms that vary in expression, localization and activity (Beck et al., 2013; Ruiz-Sola et al., 2016).

Light is one of the most important environmental factors influencing fruit pigmentation (Llorente et al., 2016a). Most of the research on the influence of light in fleshy fruits has been developed in the tomato model, characterizing key elements involved in light perception (chryptochromes and phytochromes) and signaling elements such as transcription factors PIFs (Phytochrome Interacting Factors) and HY5 (Long Hypocotyl 5), and their interactions with light signaling repressors such as COP1, CUL4-DDB1 E 3 ligase, and DET1, among others (Llorente et al., 2016b; Bianchetti et al., 2018). The current knowledge of the molecular mechanisms by which light modulates carotenoid biosynthesis and accumulation in different plant organs and species has been recently reviewed and in most cases, including tomato fruit, light stimulates carotenoid accumulation (reviewed by Llorente et al., 2017). The exploration of light regulation of carotenoid accumulation in *Arabidopsis* has revealed a direct induction of *PSY* transcription by light mediated by PIFs (Toledo-Ortiz et al., 2010) which is also coordinated with the action of HY5, conforming a dynamic activation-suppression transcriptional module in response to light and temperature cues (Toledo-Ortiz et al., 2014). It was also demonstrated that PIF1 and other photolabile PIFs promote the shade-triggered decrease in carotenoid accumulation (Bou-Torrent et al., 2015). In tomato, a PIF1 homolog (PIF1a) plays a key role in the regulation of carotenoid biosynthesis during fruit ripening by binding to the promoter of the *PSY1* and repressing carotenoid accumulation under low light or shade conditions (Llorente et al., 2016b). Interestingly, it has been proposed that this process is modulated during fruit ripening by a self-shading effect of chlorophylls since these pigments may act as light filters in green fruit tissues keeping phytochromes in the inactive form and maintaining high PIF1a level that represses *PSY1* expression. As ripening progress and chlorophyll is progressively degraded, the process is reversed, and carotenoid biosynthesis enhanced (Llorente et al., 2016b). Moreover, light also affects the transcription of βLCY in tobacco plants (Fu et al., 2013) and post-translationally controls PSY activity in tomato fruits (Schofield and Paliyath, 2005), hence light could regulate carotenoid biosynthesis through a combination of transcriptional and post-transcriptional mechanisms (Fuentes et al., 2012; Llorente et al., 2017).

In citrus fruits, color change is a developmental process that is genetically governed but, in addition, different external factors can directly affect carotenoid biosynthesis, being light and temperature key environmental determinants (reviewed by Rodrigo et al., 2013b; Lado et al., 2018). Field observations collected by growers over the years recognized a positive effect of light on peel coloration of sweet orange and mandarin fruits, since those growing inside canopy or partially covered by leaves or by other fruits on the tree (shade) display a paler peel pigmentation in comparison with a brighter orange coloration of fruit growing outside canopy directly exposed to the sunlight (Hiratsuka et al., 2012; Cronje et al., 2013; Magwaza et al., 2013; Rodrigo et al., 2013b). In different sweet orange cultivars as Tarocco, Cara Cara, and Lane Late, fruit bagging produced a faster chlorophyll degradation during ripening and reduced color intensity at maturity (Xie et al., 2013). Similar results were observed in shaded Satsuma mandarins (Yano et al., 2013). However, pigment accumulation in response to light seems to be dependent on the citrus species since in the peel of red Star Ruby grapefruit (*Citrus paradisi*) a higher accumulation of carotenes, mainly phytoene and lycopene, was registered under dark conditions (bagged or shaded), according to the development of an intense red coloration (Lado et al., 2015b). The effect of light/dark conditions on carotenoid biosynthesis and accumulation was also investigated in calli of four different citrus genotypes and the effect was highly dependent on the genotype. Consistent with data obtained in fruit, under dark conditions carotenoid content was enhanced in calli of red grapefruit but reduced in those of sweet orange (Gao et al., 2011). However, dark or shaded growing conditions decreased the expression of most carotenogenic genes in red Star Ruby grapefruit, which was not reflected by a parallel carotenoid accumulation (Lado et al., 2015b). In the flavedo of Satsuma mandarins stored under red LED light, a simultaneous increase in the expression of carotenoid biosynthetic genes and β-cryptoxanthin and other xanthophylls content was observed (Ma et al., 2012; Ma et al., 2015) and in sweet orange and red grapefruit calli an enhancement of *PSY* transcript levels by light has been reported (Gao et al., 2011). Therefore, despite the importance of peel pigmentation in the quality and marketability of citrus fruits and the relevance of light regulating this trait, information regarding the effect of light on the transcriptional regulation of carotenoids biosynthetic genes and how is related to changes in carotenoids profile in the peel of orange-colored Citrus fruits is scarce and fragmentary.

The objective of current study has been to evaluate the effect of fruit shading, in comparison with fruit subjected to standard photoperiodic conditions in the field, on carotenoid content and composition and on the expression of main carotenoid biosynthetic genes in the peel of mandarin and sweet orange fruits. The study is supplemented by an analysis of the levels of the plant hormone ABA, a downstream metabolite of the carotenoid biosynthetic pathway, in the peel of fruits submitted to those conditions. To that end, and in order to have a reliable comparison of the potential differences/similarities in carotenoid regulation among *Citrus* species and varieties, we have selected a mandarin and an orange fruit corresponding to two of the most relevant and worldwide-cultivated varieties, Clementine mandarin and Navelina orange, respectively.

## Material and Methods

### Plant Material and Treatments

Fruits of Clementine mandarins cv. Nules (Clemenules) (*Citrus clementina*) and Navelina oranges (*Citrus sinensis* L. Osbeck) growing in a commercial orchard located in Lliria (Valencia, Spain) and cultivated under standard agronomical conditions were used in this study. In a first experiment, the effect of fruit ripening stage at covering date on color development and pigment composition was carried out in Clementine mandarins. Covering experiments were essentially performed as described in Lado et al. (2015b). Mandarin fruits located in the external of tree canopy were covered with black plastic bags (leaving the bottom-end open to allow gas exchange) at three different developmental stages (July-C1; August-C2 and September-C3), coinciding with immature green (C1 and C2) to mature green (C3) stages. At monthly intervals, covered (C) and non-covered (NC) fruit were harvested and the flavedo tissue was excised, frozen in liquid nitrogen, ground to a fine powder and stored at −80º C until analysis. For each harvest time, 3 biological replicates were used consisting of 10 fruits each. Immediately after harvest, fruits were delivered to the laboratory and peel and pulp color was measured using a Minolta CR-330 colorimeter. Color is expressed as the *a/b* Hunter ratio (Stewart and Wheaton, 1972). The *a/b* ratio is negative for green fruit, the zero value corresponds to yellow fruit at color break and is positive for orange to red colored fruit. Based on results obtained in mandarin fruit, an experiment was performed Navelina sweet orange by covering the fruits at immature green stage (July). The same sampling procedure as describe for Clementine mandarin was followed throughout fruit ripening.

### Chlorophyll and Carotenoid Extraction

Flavedo (external colored layer of citrus fruit peel) pigments were extracted as previously described (Rodrigo et al., 2003). The total chlorophyll (a+b) content was determined by measuring the absorbance at 644 and 662 nm and calculated according to the Smith and Benitez equations (Smith and Benítez, 1955). After chlorophyll measurements the pigment ethereal solution was dried and saponified using a 10% methanolic:KOH solution. Free carotenoids were extracted and samples dried under N_2_ and kept at −20 ºC until analysis. All procedures were carried out on ice under dim light to prevent possible photodegradation, isomerization and structural changes of carotenoids.

### Carotenoid Analysis by HPLC-PAD

Carotenoid composition of each sample was analyzed by HPLC with a Waters liquid chromatography system equipped with a 600E pump and a model 2998 photodiode array detector (PAD), and Empower software (Waters). A C_30_ carotenoid column (250 × 4.6 mm, 5 μm) coupled to a C_30_ guard column (20 × 4.0 mm, 5 μm) (YMC, Teknokroma) was used. Samples were prepared for HPLC by dissolving the dried carotenoid extracts in CHCl_3_: MeOH: acetone (3:2:1, v:v:v). A ternary gradient elution with MeOH, water and methyl *tert*-butyl ether (MTBE) was used for carotenoid separation and gradient applied was reported in previous works (Rodrigo et al., 2003; Carmona et al., 2012). Carotenoids were identified by comparison of the spectra and retention time with those of authentic standards, when available, or by matching the observed versus literature spectral data and retention time under identical chromatographic conditions (Britton, 1995; Rouseff and Raley, 1996; Rodrigo et al., 2003). The carotenoid peaks were integrated at their individual maxima wavelength and their content was calculated using calibration curves of β-cryptoxanthin (Extrasynthese), lutein (Sigma), anteraxanthin (CaroteNature), violaxanthin (CaroteNature) for violaxanthin and neoxanthin isomers, zeaxanthin (Extrasynthese), β-carotene (Sigma) for α- and β-carotene, and β-apo-8′-carotenal (Extrasynthese) for β-citraurin. Phytoene, phytofluene and ζ-carotene were previously purified by thin layer chromatography from carotenoid extracts of Pinalate orange fruit and the corresponding calibration curves performed (Rodrigo et al., 2003; Lado et al., 2015b). The concentration of individual carotenoids are expressed as micrograms per grams of sample on fresh weight basis (FW). The three biological replicates were extracted twice and each analytical determination was replicated at least twice.

### Quantitative Real Time-PCR

Total RNA was isolated from the fruit flavedo at each harvesting date, using RNeasy Plant Mini Kit (Qiagen) and subsequently treated with DNase (DNA free, DNase treatment & removal, Ambion). The transcripts present in 2 μg of total RNA were reverse-transcribed using SuperScript III Reverse Transcriptase (Invitrogen) in a total volume of 20 μL. One microliter of a 5 times diluted first-strand cDNA, containing approximately 100 ng of cDNA, was used for each amplification reaction. Quantitative real-time PCR was performed on a LightCycler 480 instrument (Roche), using the LightCycler 480 SYBRGreen I Master kit (Roche) and following the manufacturer’s instructions. The genes analyzed related to carotenoid precursors, biosynthesis and catabolism were: 1-Deoxy-D-xylulose-5-phosphate synthase (DXS1), hidroxymethylbutenyl diphosphate reductase (HDR), geranyl geranyl pyrophosphate synthase (GGPPS1), phytoene synthase (PSY), phytoene desaturase (PDS), ζ-carotene desaturase (ZDS1), β-lycopene cyclase 1 (βLCY1), β-lycopene cyclase 2 (βLCY2a and βLCY2b), β-carotene hydroxylase (βCHX) and carotenoid cleavage diosxigenase (CCD4b1). The primers employed for the amplification of each gene have been previously described in Lado et al. (2015b) and Rodrigo et al. (2013b). The cycling protocol, for all genes analyzed, consisted of 10 min at 95°C for pre-incubation, 40 cycles of 10 s at 95°C for denaturation, 10 s at 59°C for annealing and 10 s at 72°C for extension. Fluorescent intensity data were acquired during the extension time. Specificity of the PCR reaction was assessed by the presence of a single peak in the dissociation curve performed after the amplification steps. For expression measurements, we used the LightCycler 480 Software release 1.5.0, version 1.5.0.39 (Roche) and calculated expression levels relative to values of a reference sample using the Relative Expression Software Tool (Pfaffl et al., 2002). Normalization was performed using the expression levels of the *actin* gene (Alós et al., 2014). For all genes and varieties analyzed, the reference sample was the expression value obtained in the flavedo of fruits harvested in July (immature green stage). Results were the average of 4 analyses for each of the 3 biological replicates used. To test for significant differences on gene expression between NC and C fruit in each harvest date a pair wise fixed reallocation randomization test was applied (p ≤ 0.05).

### ABA Quantification

Analysis was carried out by HPLC-MS/MS as described before (Lado et al., 2015b). Briefly, 0.4 g of fresh fruit peel was spiked with 100 ng of [^2^H_6_]-ABA and extracted in 5 mL of distilled water. Extract were then centrifuged at 4 ºC, supernatants were recovered and pH adjusted to 3.0 with acetic acid (30%). The acidified extract was partitioned twice against 3 ml of diethyl-ether. The organic layers were evaporated under vacuum and the dry residue was then re-suspended in MeOH (10%) solution. The resulting solution was filtered and injected into a HPLC system (Waters Alliance 2695, Milford, MA, USA). A Kromasil 100 C18 column (100 × 2.1 mm, 5 μm particle size; Scharlab, Barcelona, Spain) was used for ABA separation using a linear gradient of MeOH and water (0.01% acetic acid) at a flow rate of 300 μl min^−1^. Quantification was performed with triple quadrupole mass spectrometer (Quattro LC, Micromass Ltd., Manchester, UK) connected online to the output of the column through an orthogonal Z-spray electrospray ion source. Two independent determination for each of the 3 biological replicates were done. Significant differences between NC and C fruit at each harvest date, a two-tailed unpaired Student’s *t*-test was applied (*P* ≤ 0.05).

## Results

### Fruit Shading Affected Color Development and Pigment Accumulation in the Peel of Clementine Mandarin

**Figure 1:**
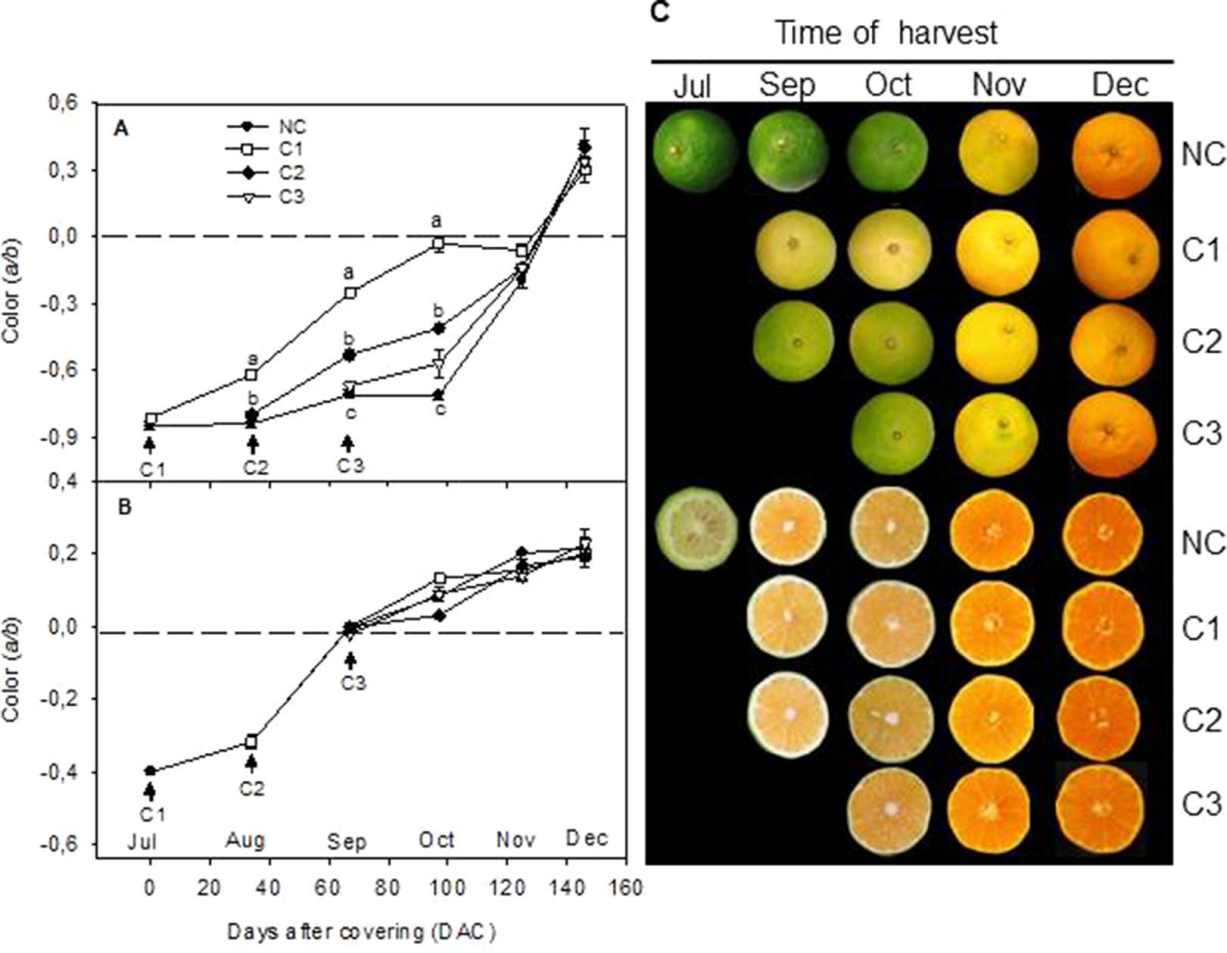
Effect of shading time on color (*a/b* Hunter units) of the peel **(A)** and pulp **(B)** and external and internal appearance **(C)** of Nules Clementine mandarin during ripening. Arrows indicate three different covering times (C1-July, C2-August, and C3-September). Fruits were harvested at monthly intervals from September to December Letters indicate significant differences between NC and C fruit for each harvest date determined by Tukey test (p ≤ 0.05).

In order to explore the effect of light on color development and carotenoids biosynthesis and accumulation in Citrus, green fruits of Clementine mandarin were covered at three different developmental stages: July (C1), August (C2), and September (C3). Color development, and accumulation of chlorophyll (Chl) and carotenoids were followed in covered and non-covered control fruit (NC) during the ripening period up to end of December. In NC fruits, the *a/b* Hunter ratio of the peel remained almost constant until October when it progressively reached the breaker stage in November and values corresponding to orange pigmentation (0.41 ± 0.08) in December (**Figures 1A, C**). Interestingly, light avoidance accelerated mandarin peel degreening and the earlier the fruits were covered the faster coloration took place. Then, more rapid peel degreening occurred in the fruit covered in July (C1) compared to fruits covered in August (C2) or September (C3) (**Figure 1A**). This effect of shading was maintained until October and thereafter, no differences in the color of the peel were observed among the different experiments. Then, in December C and NC fruits reached commercial maturity and similar peel coloration (**Figures 1A, C**). Fruit shading, however, did not affect the rate or the final coloration of the pulp (**Figures 1B, C**), suggesting that the effect of light avoidance on coloration in mandarin fruits is restricted to the peel. Then pigment analysis and expression of carotenoid biosynthetic genes were only performed in the peel.

**Figure 2:**
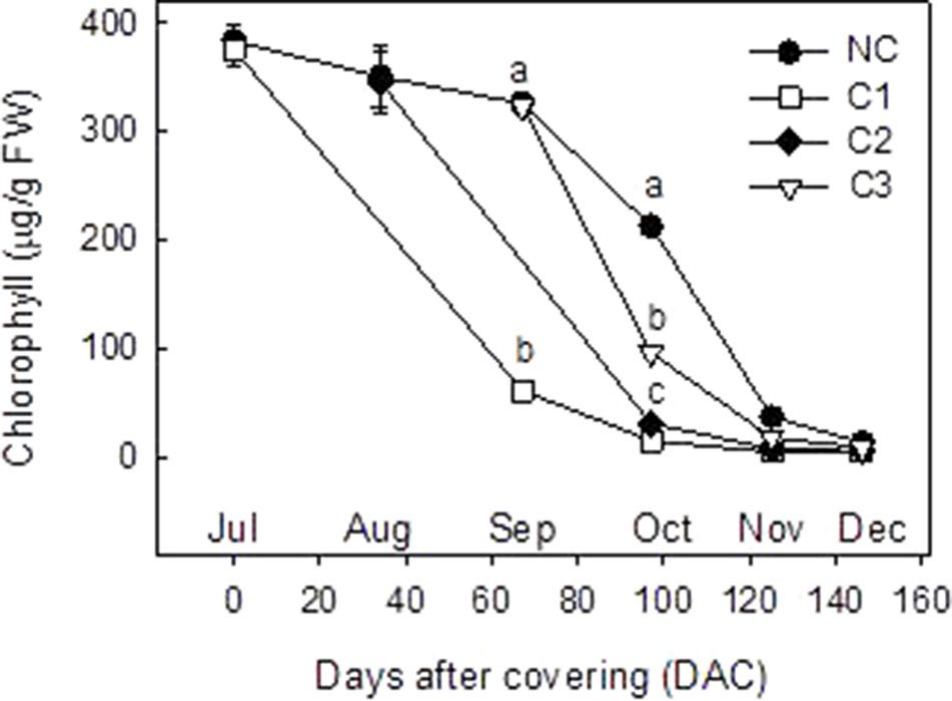
Total chlorophyll content (µg/g FW) in the peel of non-covered fruit and of fruits covered at three times (C1-July, C2-August, and C3-September). The data are means of at least two analyses for each of the 3 biological replicates ± SE. Letters indicate significant differences between NC and C fruit in each harvest date determined by Tukey test (p ≤ 0.05).

**Figure 3:**
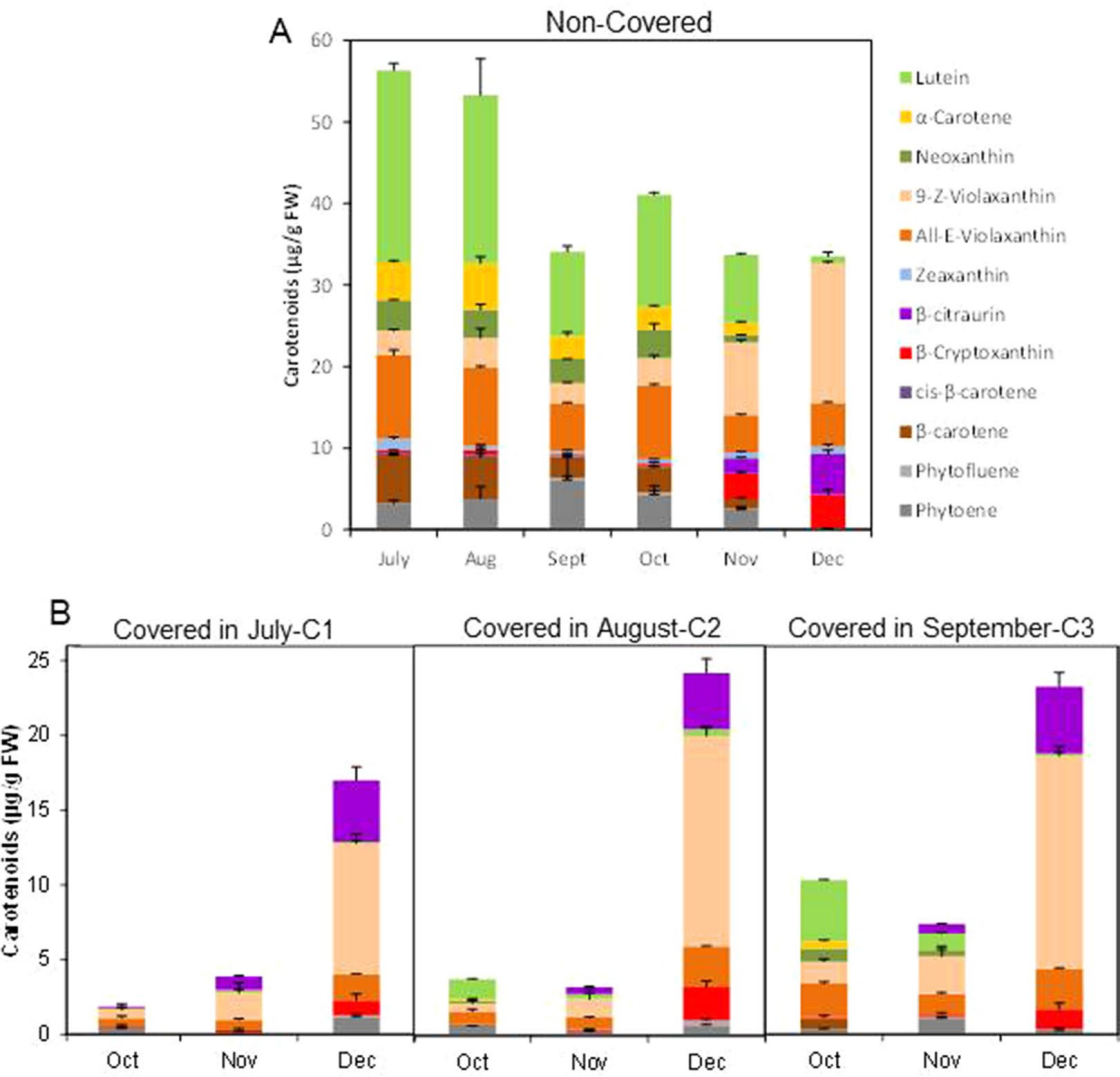
Carotenoid content and composition (µg/g FW) in the peel of non-covered (NC) **(A)** and covered (C) fruits covered at three times (C1-July, C2-August, and C3-September) **(B)**. The data are means of at least two analyses for each of the 3 biological replicates ± SE.

Analysis of Chl and carotenoid content were performed in the peel of C and NC Clementine mandarin from July to December (**Figures 2** and **3**). In control NC fruits, total Chl remained at high levels (380 µg/g FW) until September, when experienced a progressive decrease to reach very low levels in December (13.2 ± 2.3 µg/g FW). Fruit shading at the 3 developmental stages accelerated the rate of Chl degradation in the peel. Thus, Chl content in September in C1 fruits was 3-times lower than in control fruits, and in October the content of Chl in the peel of covered fruits was also lower than in non-covered. At latter stages of ripening (after November) Chl almost disappeared in the peel of the fruits and also differences among treatments (**Figure 2**).

Light avoidance during fruit ripening caused striking modifications in carotenoid content in the peel of Clemenules mandarin. In NC fruits, total carotenoids decreased from July to September (56.3 ± 2.5 to 34.1 ± 0.98 µg/g FW), slightly increased in October to become stable afterwards (30–40 µg/g FW) (**Figure 3A**). Fruit bagging produced a marked reduction in total carotenoids, and the magnitude was depended on the covering date (**Figure 3B**). In early covered fruit (July C1), total carotenoids decreased almost 30-times in the next two months, resulting in October about 20-times lower than control fruits. As fruit bagging was delayed differences in carotenoid content were less dramatic with respect to untreated fruits, since in October carotenoids were reduced by 84% and 43.5% in C2 and C3 fruits, respectively. Interestingly, carotenoids content in the peel of shaded fruit experienced a marked upsurge (4-, 7- and 3-times over November) at commercial harvest (December), even the total content remained still lower than in control fruit (**Figures 3A, B**).

The composition of individual carotenoids was also affected by light avoidance. The peel of control fruits at the 3 shading times (July, August, and September) contained the typical carotenoids of chloroplastic tissues: lutein (10.3 to 23.5 µg/g FW), *9-Z*- and *all-E*-violaxanthin (8.3 to 13.2 µg/g FW), α- and β-carotene (5.4 to 10.8 µg/g FW), neoxanthin, and zeaxanthin (0.5 to 1.4 µg/g FW) as well as the colorless phytoene (3.2 to 6.2 µg/g FW) (**Figure 3A**). As ripening progressed, the concentration of chloroplastic carotenoids decreased and increased that of violaxanthin (*9-Z*- and *all-E*-) to reach levels as higher as 22.4 µg/g FW in December, and β-cryptoxanthin and the apocarotenoid β-citraurin, which are the characteristic pigments present in the peel of full mature mandarins (**Figure 3A**).

Light avoidance not only reduced the concentration of total carotenoids but also modified their composition. The concentration of chloroplastic carotenoids was more markedly reduced as early as the covered time. Moreover, small amount of phytoene was detected in all shaded fruits during ripening. Interestingly, accumulation of the apocarotenoid β-citraurin was initially detected at the same time than in control fruits, and it was less affected by shading than other carotenoids. Fruits growing in the dark were almost devoid of zeaxanthin, which was present in all ripening stages in control fruits. It is worth to mention that in shaded fruits in December, after the upsurge of carotenoids, the profiling and distribution of carotenoids were similar to that of non-covered fruits (with the exception of zeaxanthin), represented mainly by violaxanthin (mostly *9-Z*- isomer), β-citraurin, and β-cryptoxanthin (**Figures 3A–C**).

### Fruit Shading Reduced Peel Coloration and Carotenoid Content in the Peel of Navelina Oranges

**Figure 4:**
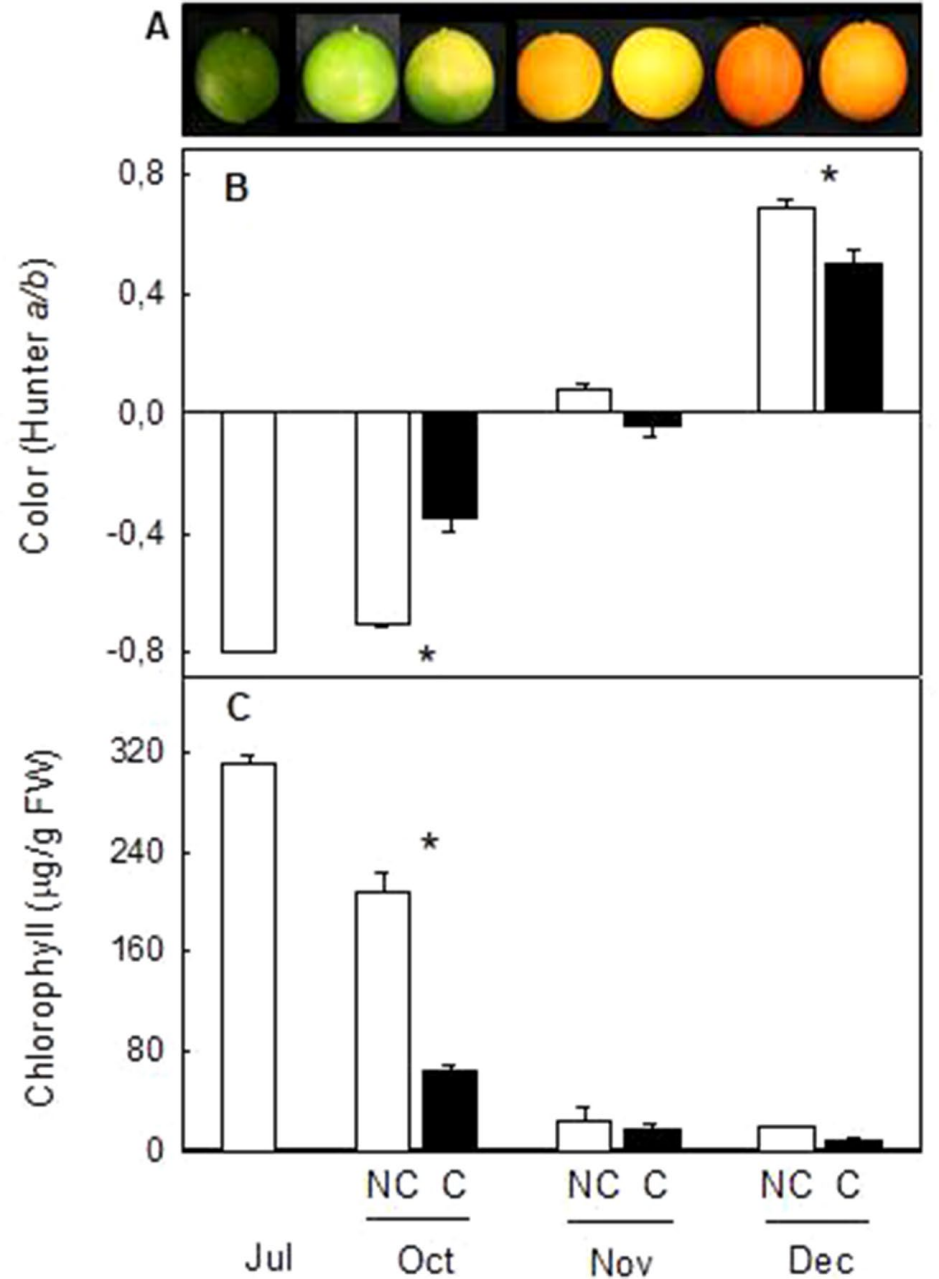
Effect of fruit shading on external appearance **(A)**, peel color (*a/b* Hunter) **(B)** and total chlorophylls content **(C)** in the peel of non-covered (NC) and covered (C) Navelina fruits. Fruit were covered in the field in July and harvested from October to December. Asterisks indicate significant differences between NC and C fruit for harvest date determined by T unpaired test (p ≤ 0.05).

In order to confirm the effect of light avoidance on peel coloration and carotenoid biosynthesis and extend the conclusion to fruits of other Citrus genotypes with different pattern of carotenoid accumulation, fruit shading experiments were performed in Navelina sweet orange (*C. sinensis* L.). This orange cultivar was selected by the bright coloration of the peel, the ripening time (middle season) and because it is an orange cultivar of high production in Spain. Immature-green fruit were covered in July, since it was the time of major effects in fruit of Clementine mandarin (**Figure 1**). Similar to Clementine mandarins, fruit bagging accelerated peel degreening but also reduced orange coloration in mature fruits (**Figure 4**). Two month after covering, Chl content of the peel was substantially lower and the a/b ratio higher in covered than in non-covered fruits. As fruit ripening progressed, fruit exposed to the light developed a more intense orange coloration than covered fruit, with significant higher a/b values (0.68 ± 0.02 and 0.50 ± 0.04, in non-covered and covered fruits, respectively) (**Figure 4**).

**Figure 5:**
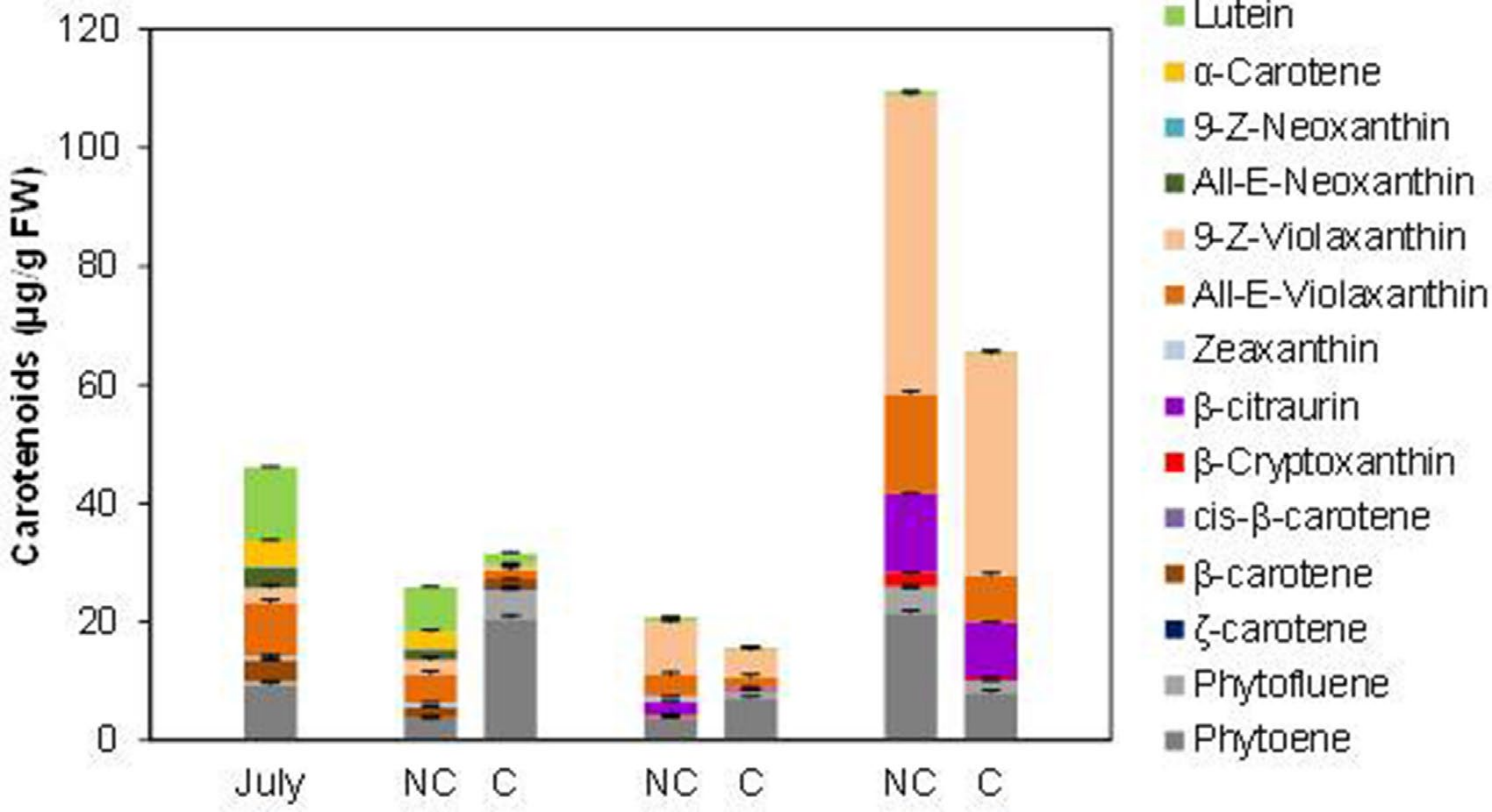
Carotenoid content and composition (µg/g FW) in the peel of non-covered (NC) and covered (C) Navelina fruits. Fruit were covered in the field in July and harvested from October to December. The data are means of at least two analyses for each of the 3 biological replicates ± SE.

Total carotenoids content in the peel of Navelina at the shading time was moderated (46.0 µg/g FW) with a profiling typical of chloroplastic tissues (26.9% lutein, 25% violaxanthin, 20.4% phytoene, 17.4% α- and β-carotene, and 9.1% zeaxanthin and neoxanthin) (**Figure 5**). During fruit degreening and initial color development, total carotenoids decreased in the peel of control fruits and only slight quantitative differences over covered fruits were observed. However, the distribution of individual carotenoids was substantially altered by shading. Then, in C fruit chloroplastic carotenoids decreased faster than in control fruit and accumulated large amount of colorless carotenes (phytoene and phytofluene), accounting 80% of total carotenoids in October compared to 15% in NC fruit (**Figure 5**). The presence of β-carotene (0.31 µg/g FW) was also detected only in the peel of C fruits three months after covering. As ripening progressed, linear carotenes were reduced and started the accumulation of xanthophylls that experienced a major increment in mature fruits at December. Total carotenoids content was higher (68%) in control than in NC fruits, but the distribution of individual carotenoid was slightly different. The *9-Z* and *all-E*-violaxanthin isomers, β-citraurin and β-cryptoxanthin accounted 76% of total carotenoids in NC fruits while linear carotenes (phytoene+phytofluene) were 24%. In shaded (C) fruits, the distribution was 60% xanthophylls and 40% linear carotenes. It is noteworthy that the concentration of the apocarotenoid β-citraurin was similar in both type of fruit (12% in NC and 14% in C fruits), suggesting that the synthesis of this compound is not affected by shading (**Figure 5**).

### Fruit Shading Downregulates the Expression of Carotenoid Biosynthetic Genes in the Peel of Clementine Mandarin and Navelina Orange

**Figure 6:**
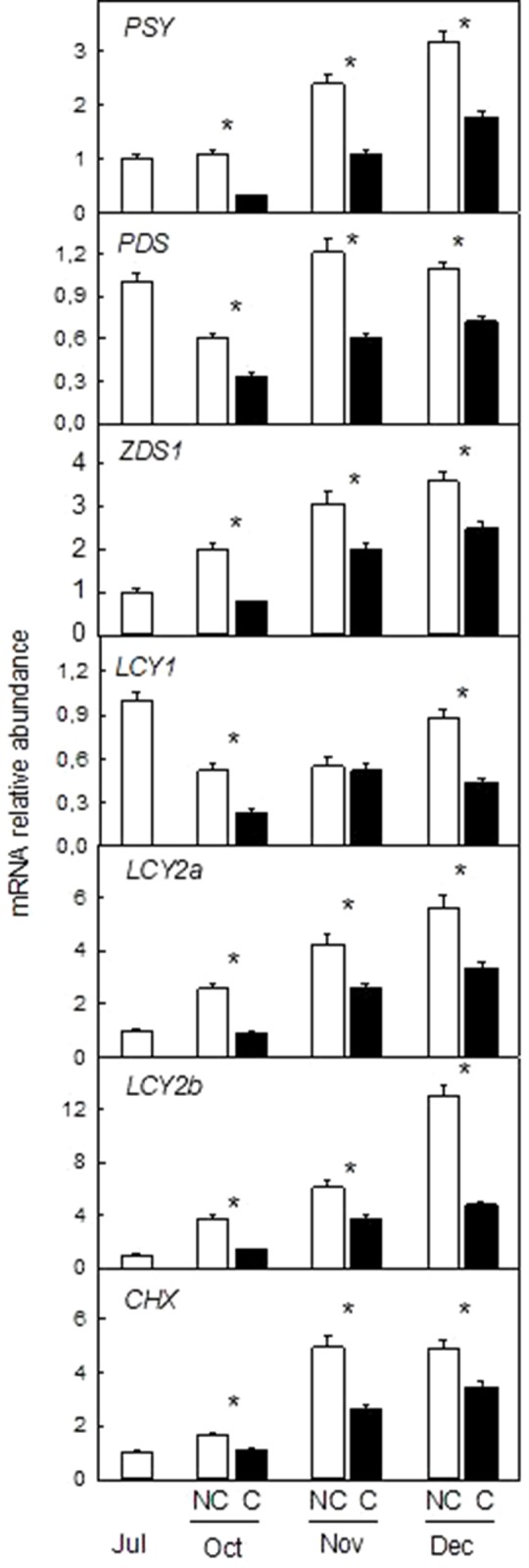
Effect of shading on the expression of carotenoid biosynthetic genes in the peel of non-covered (NC) and covered (C) Nules Clementine mandarin fruits. Fruits were covered in July (C1) and harvested from October to December. The genes analyzed were: *phytoene synthase* (*PSY*), *phytoene desaturase* (*PDS*), ζ-carotene *desaturase-1* (*ZDS1*), *β-lycopene cyclase 1* and *2a, b* (*LCY1*, *LCY2a*, and *LCY2b*), *β-carotene hydroxylase* (*CHX*). The data are means of four determinations for each of the 3 biological replicates ± SE. Asterisks indicate significant differences between C and NC fruit for each harvest date determined by the pair wise fixed reallocation randomization test (p ≤ 0.05).

**Figure 7:**
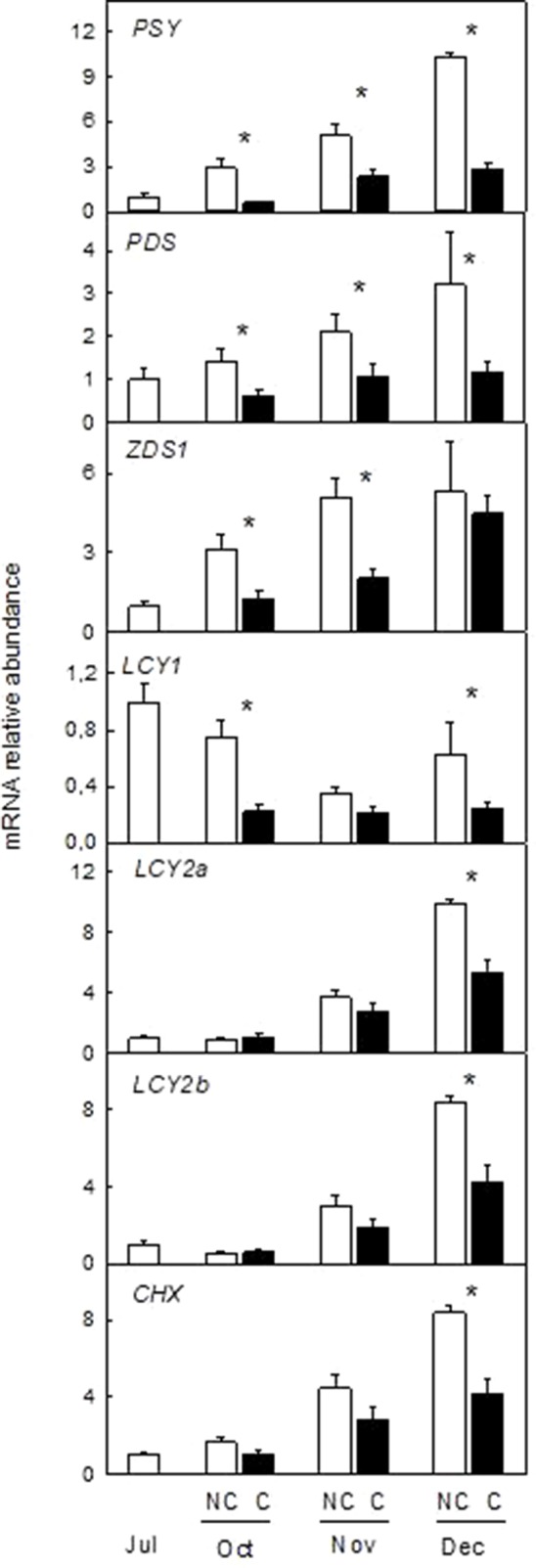
Effect of shading on the expression of carotenoid biosynthetic genes in the peel of non-covered (NC) and covered (C) Navelina orange fruits. Fruits were covered in July (C1) and harvested from October to December. The genes analyzed were: *phytoene synthase* (*PSY*), *phytoene desaturase* (*PDS*), ζ-carotene *desaturase-1* (*ZDS1*), *β-lycopene cyclase 1*, and *2a,b* (*LCY1*, *LCY2a*, and *LCY2b*), *β-carotene hydroxylase* (*CHX*). The data are means of four determinations for each of the 3 biological replicates ± SE. Asterisks indicate significant differences between C and NC fruit for each harvest date determined by the pair wise fixed reallocation randomization test (p ≤ 0.05).

To decipher if the changes in peel color and carotenoid accumulation in response to shading were associated with differences in the expression of carotenoid biosynthesis genes, transcript levels of *PSY*, *PDS*, *ZDS1*, *LCY1*, *LCY2a*, *LCY2b*, and *CHX* were evaluated in the peel of NC and C Clementine mandarin (**Figure 6**) and Navelina oranges (**Figure 7**).

In the peel of light-exposed Clementine fruits, the expression of all the genes analyzed was higher than in the peel of C fruits and in both cases transcript abundance increased during ripening (**Figure 6**). With the exception of *PDS* and *LCY1* that were not affected or reduced, accumulation of the other transcripts experienced an important increase during ripening in light-exposed Clementine mandarins (from 3-times PSY to 13-times *LCY2a*). Absence of light reduced accumulation of all the transcripts as early as in October (around 50% reduction over NC fruits). This effect was maintained until the ripen stage and in December accumulation of transcripts of the seven genes analyzed were lower in C than in NC fruits (**Figure 6**).

Downregulation of carotenoid biosynthetic genes by absence of light was also confirmed in fruits of Navelina orange (**Figure 7**). In light-exposed fruits, transcript accumulation of most genes (with the exception of *LCY1*) increased substantially over ripening. Interestingly, transcript accumulation was also enhanced during ripening of C fruits but to a lower extent than in NC fruits. Thus, in December abundance of the *PSY* transcript was reduced by 4-times while those of *PDS*, *LCYY1*, *LCY2a*, *LCY2b*, and *CHX* were 50% lower in C than in NC fruits (**Figure 7**). These results suggest that absence of light repress but not suppress the natural induction of carotenoid biosynthetic genes.

### Effect of Fruit Shading on the Expression of Genes of the Carotenoid Precursor (MEP Pathway) and Catabolism

**Figure 8:**
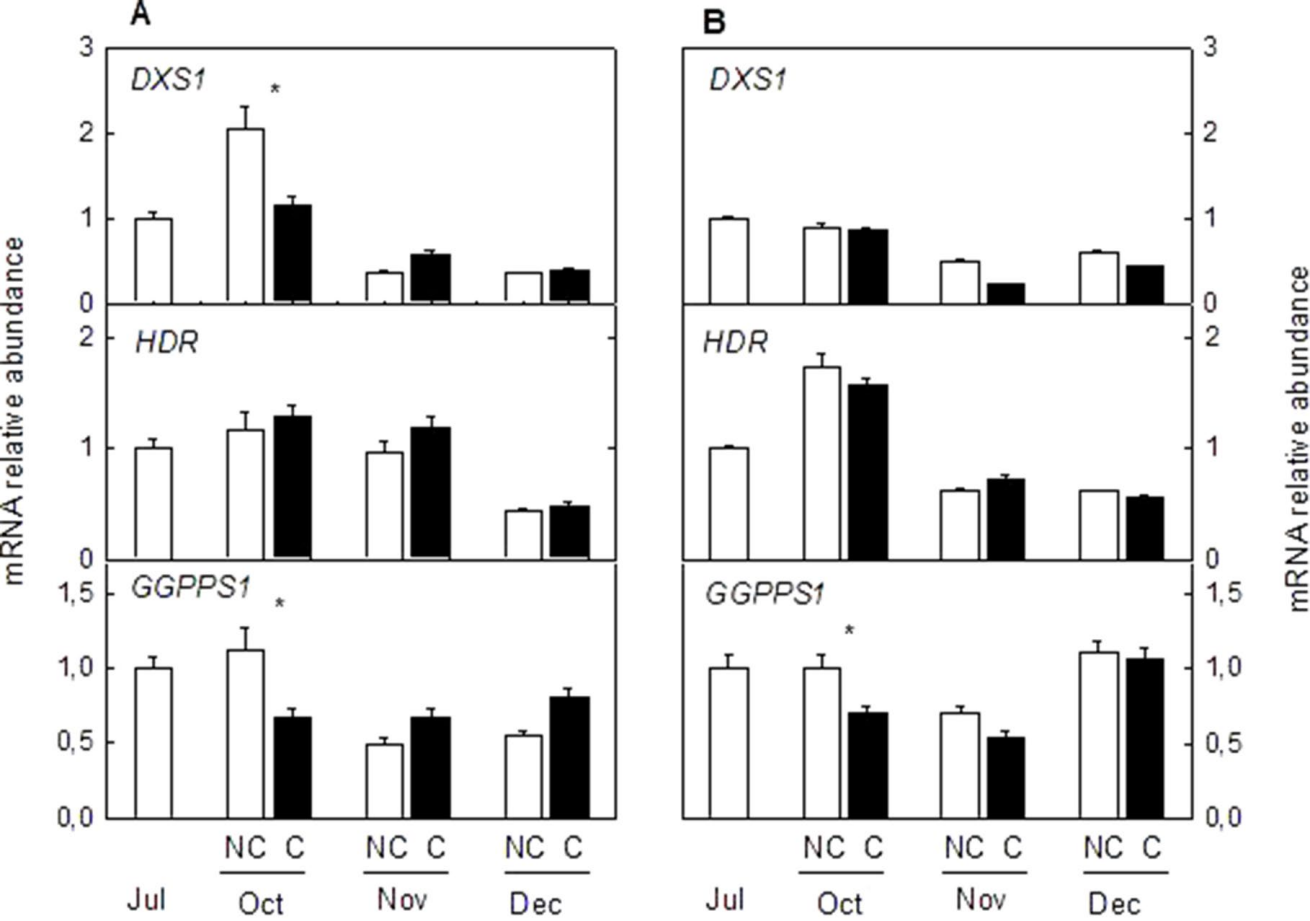
Effect of shading on the expression of Methylerythritol 4-phosphate pathway (MEP) genes in the peel of non-covered (NC) and covered (C) Clementine **(A)** and Navelina orange **(B)** fruits. Fruits were covered in July (C1) and harvested from October to December. The genes analyzed were: *1-deoxy-D-xylulose-5-phosphate synthase* (*DXS*), *4-hydroxy-3-methylbut-2-enyl diphosphate reductase* (*HDR1*), and *geranyl diphosphate synthase* (*GGPPS1*). The data are means of four determinations for each of the 3 biological replicates ± SE. Asterisks indicate significant differences between NC and C fruit in each harvest date determined by the pair wise fixed reallocation randomization test (p ≤ 0.05).

**Figure 9:**
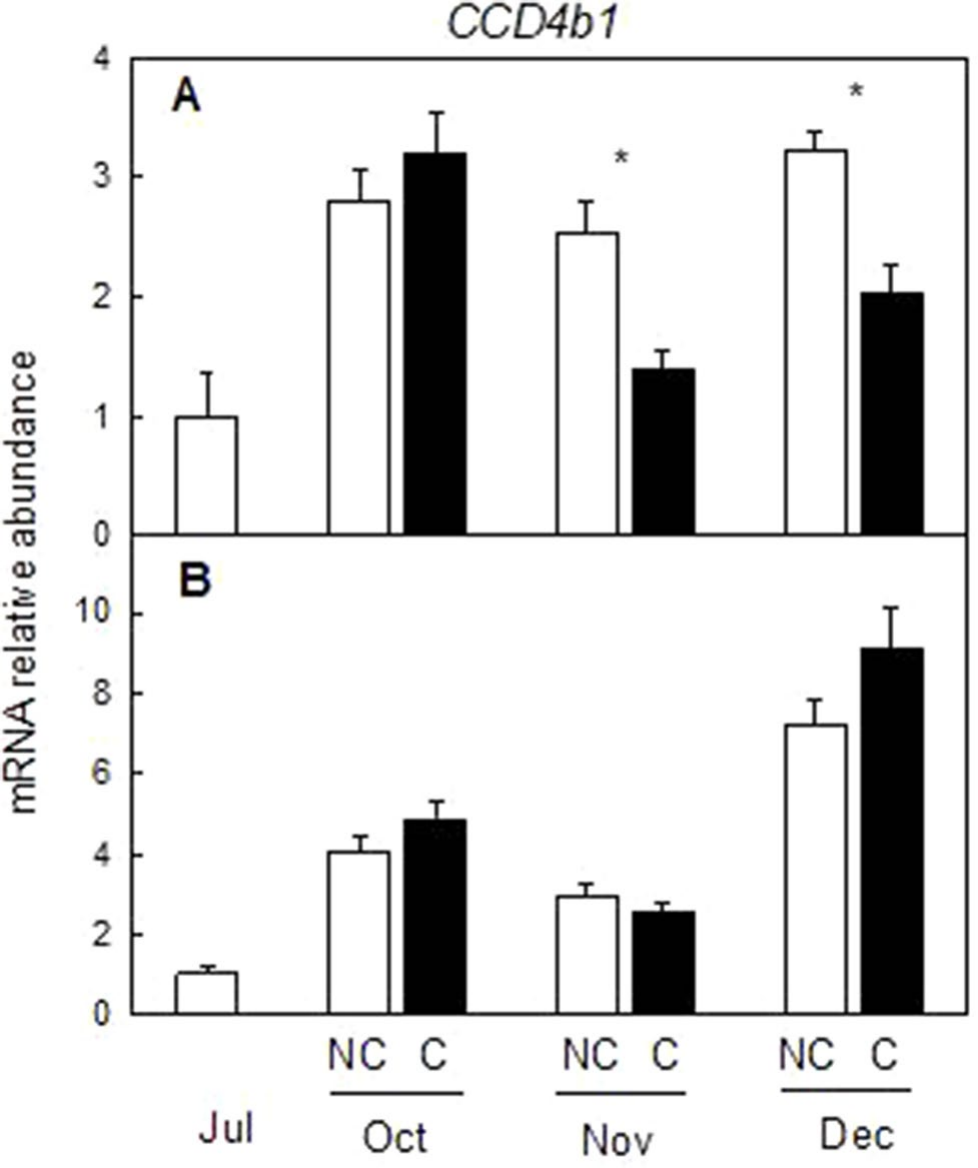
Effect of shading on the expression of *carotenoid cleavage dioxygenase 4b1* (*CCD4b1*) gene in the peel of non-covered (NC) and covered (C) Clementine **(A)** and Navelina orange **(B)** fruits. Fruits were covered in July (C1) and harvested from October to December. The data are means of four determinations for each of the 3 biological replicates ± SE. Asterisks indicate significant differences between NC and C fruit in each harvest date determined by the pair wise fixed reallocation randomization test (p ≤ 0.05).

To determine if other pathways related to carotenoid metabolism may be also affected by fruit shading, accumulation of transcripts corresponding to three genes (*DXS*, *HDR1*, and *GGPPS1*) involved in the synthesis of carotenoids precursors of the MEP pathway (**Figure 8**) and one carotenoid cleavage dioxygenase gene (CCD4b1) (**Figure 9**) involved in the synthesis of the apocarotenoid β-citraurin, were analyzed in the peel of C and NC fruit of Clementine mandarin and Navelina orange. In general, accumulation of the three transcripts of the MEP pathways did not experience important alterations during ripening of light-exposed fruits. Fruit shading did not modify accumulation of the corresponding mRNAs neither in mandarin nor oranges (**Figure 8**). The expression of CCD4b1 significantly increased at early stages of ripening (October) in mandarin and orange fruit, and a major enhancement was also detected in late-mature oranges (December). Fruit shading only reduced the expression of *CCD4b1* in middle-season Clementine fruits but it did not alter accumulation of the transcript in orange fruit during the complete ripening period (**Figure 9**).

### ABA Content in the Peel of Clementine Mandarin and Navelina Oranges Was Unaffected by Fruit Shading

**Figure 10:**
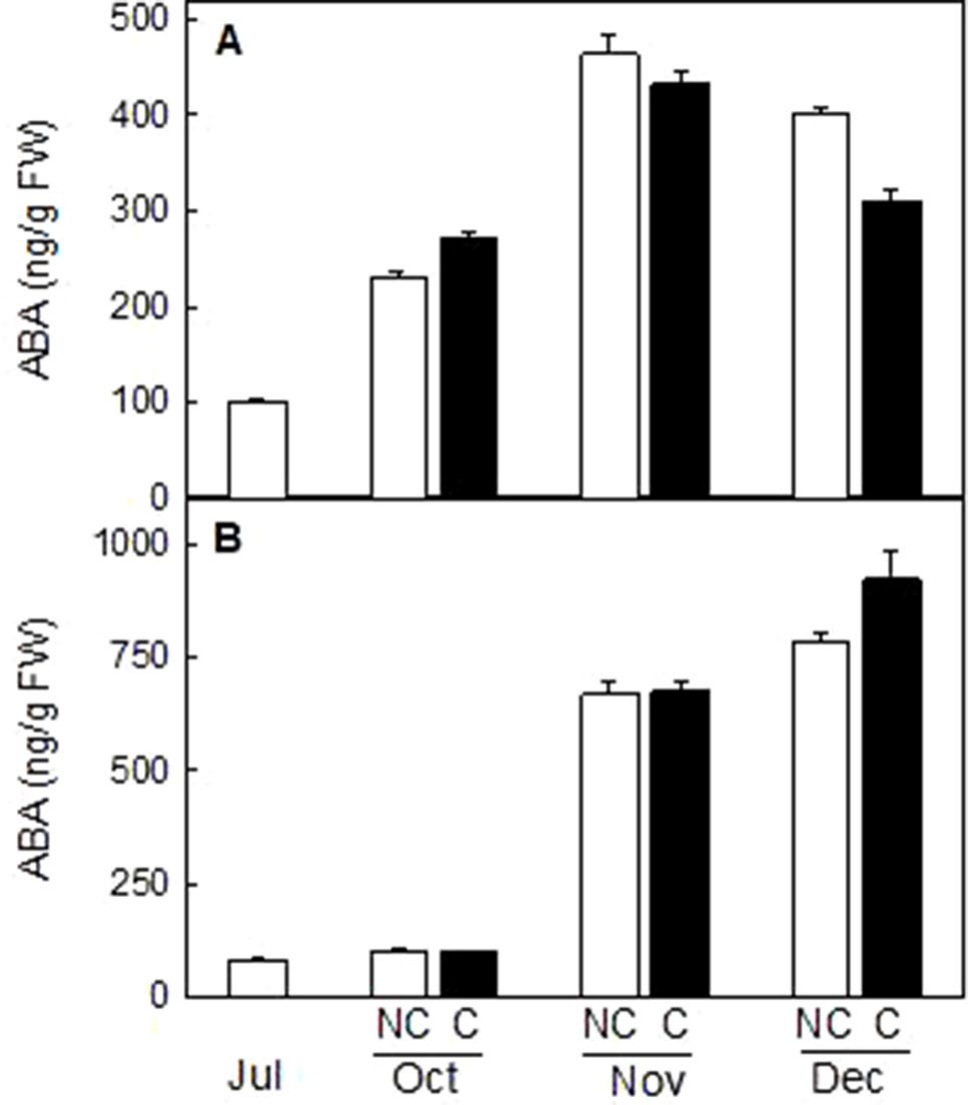
Effect of shading on ABA content (ng/g FW) in the peel of non-covered (NC) and covered (C) Clementine **(A)** and Navelina orange **(B)** fruits. Fruits were covered in July (C1) and harvested from October to December. The data are means of two determinations for each of the 3 biological replicates ± SE. No significant differences were detected between NC and C fruit determined by T unpaired test (p ≤ 0.05).

The content of the plant hormone ABA, a C-15 apocarotenoid downstream in the carotenoid biosynthetic pathway, was determined in the peel of NC and C mandarin and orange fruits. ABA content increased during ripening in both varieties, reaching a maximum in mandarins at mid-ripening (464 ± 19 ng/g FW in November) and in full-mature fruits in oranges (922 ± 66 ng/g FW in December). It is worth noting that fruit shading did not produce important differences in ABA content in the peel of either mandarin or orange fruits (**Figure 10**).

## Discussion

Light is probably one of the major environmental factors influencing coloration of fleshy fruits and their commercial quality (Llorente et al., 2016a). In Citrus, external fruit coloration is a key attribute determining quality standards of the market and consumer acceptance (Lado et al., 2018). It has long been observed that fruits growing outside the tree canopy and directly exposed to the sunlight develop a brighter orange pigmentation than shaded-fruits located inside the canopy. Moreover, the peel of inside fruits presented lower ascorbic acid content (Lado et al., 2015a), phenolic, carbohydrate and total carotenoids compared to light-exposed fruits (Cronje et al., 2013; Magwaza et al., 2013). In the present manuscript, we show that deprivation of light to mandarin and orange fruits accelerated peel degreening, reduced carotenoid accumulation and downregulated the expression of carotenoid biosynthetic genes. In red grapefruit, light avoidance also accelerated chloroplast degradation but induced accumulation of the red carotene lycopene, generating fruit with red peel coloration (Lado et al., 2015b). It was suggested that light might interfere with the developmental-regulated differentiation of the chromoplasts accumulating lycopene, since accumulation of this carotene in the peel is a very unusual feature in Citrus fruits (Lado et al., 2015c; Llorente et al., 2017).

Changes in fruit pigmentation during maturation, and the transition from chloroplast to chromoplats, is the result of both chlorophyll degradation and carotenoid accumulation (Rodrigo et al., 2013a). In light-exposed mandarins, chlorophyll degradation was initiated around October (**Figure 2**) and progressively advanced in coordination with a slow reduction of chloroplastic carotenoids and the increment in colored xanthophylls (**Figure 3**). Fruit maturation under shading conditions resulted in an advanced color change in the peel of both Clemenules mandarin (**Figure 1**) and Navelina oranges (**Figure 4**), but Chl breakdown and carotenoid accumulation were differentially affected. After fruit shading, chlorophyll was rapidly degraded independently of the covering date and fruit coloration enhanced (**Figures 2** and **4**). Moreover, chloroplastic carotenoids, which were abundant in the peel of immature-green fruits, were also rapidly transformed, and, consequently, covered fruits of both species in October and November developed a yellowish appearance since were almost devoid or with very low carotenoids. The magnitude of these responses was more pronounced in Clementine mandarin than in oranges, indicating that probably differences in carotenoid content and complement between species may determine the influence of light or the response to its deprivation (**Figures 3** and **5**). Altogether, these effects resemble those occurring during dark-induced senescence of vegetative tissues (Brouwer et al., 2012; Liebsch and Keech, 2016) and then it is likely that a primary physiological consequence of fruit shading in green flavedo cells of Citrus fruits is an accelerated senescence and the disruption of the normal photo-morphogenetic transition from chloroplast to chromoplats (Llorente et al., 2016a).

The time of fruit shading appears to be also critical for the accumulation of carotenoids in the peel of Clementine mandarins (**Figure 3**). A drastic reduction in total carotenoids were registered in the peel of mandarins covered in July and August, that remained at low levels (less than 10% respect to light-growing fruits) as later as November. However, in fruit covered in September this reduction was of less intensity. At commercial harvest (December), the concentration of total carotenoids in shaded fruits was reduced between 50 and 35% with respect to non-covered fruits. Thus, the sooner the fruits were shaded, the larger was the reduction in carotenoid content. This assumption is consistent with previous observations in fruit of other *Citrus* species and varieties in which early shading reduced coloration and carotenoid content (Hwang et al., 2004; Xie et al., 2013; Yano et al., 2013). These results suggest that an early deprivation of the light signaling may affect chloroplast morphogenesis that would interfere the natural induction of peel pigmentation. The temporal analysis of carotenoid accumulation and of the expression of carotenoid biosynthesis genes in fruits of Citrus species with contrasting peel coloration indicate that stimulation of the peel color changes occur early in the maturation program and before the appearance of visible pigmentation, around October in countries of the north hemisphere (Ikoma et al., 2016; Rodrigo et al., 2013a; Wei et al., 2017). Then, it is reasonable that a latter (September) deprivation of the light signaling would have less incidence in carotenoid accumulation.

Stimulation of carotenoid accumulation by light has been documented in fruits of many species, such as tomato (Alba et al., 2000; Gupta et al., 2014; Llorente et al., 2016b), mango (Wu et al., 2013), and persimmon (Hu et al., 2013). In *Citrus*, light-exposed mandarins had higher carotenoids and external coloration than inside fruits (Cronje et al., 2013; Magwaza et al., 2013). In addition, an increase in total carotenoid and specifically in β-cryptoxanthin has been reported in the peel of Satsuma mandarins exposed to red LED light (Ma et al., 2012; Ma et al., 2015) and juice sacs from mandarins and oranges exposed to blue light (Zhang et al., 2015). In the current study, we found that both Clementine mandarin and Navelina orange growing under shading conditions experienced a marked increase in carotenoids at late maturity (December) and that their carotenes and xanthophylls proportion is similar to that of light-exposed fruits (**Figures 3** and **5**). These results may be unexpected, since absence of light originated white tomato fruits devoid of carotenoids (Cheung et al., 1993) or with reduced lycopene content (Fantini et al., 2013). Then, we suggest that a light-independent carotenoid stimulation may operate in the peel of *Citrus* fruit at advanced stages of maturity, probably developmentally regulated. This situation is not unexpected in Citrus, since the synthesis and accumulation of carotenoids in the pulp of Citrus fruits, that in general is lower than in the peel, occur in complete absence of light (Alquézar et al., 2008; Wei et al., 2017). Therefore, we hypothesize that regulation of carotenogenesis in Citrus fruits may respond to a light-dependent system that is operative in the peel, which is the part of the fruit usually exposed to the environment. A second light-independent factor, that is still known, might be responsible for the regulation of carotenoids in the pulp during the whole maturation and potentially also in the peel under prolonged dark periods.

Despite the concentration of total carotenoids in mature shaded fruits (December) was lower than that of light-grown fruits, the content of the apocarotenoid β-citraurin was similar in both type of fruits. This C_30_-apocarotenoid is the product of the cleavage of zeaxanthin and β-cryptoxanthin by the carotenoid cleavage dioxygenase CCD4b1 and the responsible of the intense orange–reddish coloration of the peel of oranges and mandarins (Ma et al., 2013; Rodrigo et al., 2013b). The presence of similar amounts of β-citraurin in the peel of covered and non-covered Clementine mandarins may explain the fact that the color of the peel in fruits harvested in January was almost the same despite the important differences in total carotenoids (**Figures 1** and **3**). In mature Navelina oranges, however, the reduction in the concentration of the β-citraurin and in β-cryptoxanthin may justify the lower coloration of the peel of covered fruits (**Figure 4**). These results corroborate the motion that the apocarotenoid β-citraurin is a major contributor to the external color of Citrus fruits (Rodrigo et al., 2013b). The up-regulation of *CCD4b1* gene in the peel of both orange and mandarin fruits occurred earlier (October) than the major accumulation of the apocarotenoid (November), in agreement with previous results (Rodrigo et al., 2013b), but in general, it was very similar in covered and non-covered fruits (**Figure 9**). These observations indicate that the expression of the *CCD4b1* in the peel of Citrus fruits appears not to be modulated by light/dark conditions, then contributing to the accumulation of β-citraurin and peel coloration under shading growing conditions. The expression of a *CCD4b1* gene, involved in the catabolism of β-carotene, is promoted during *Arabidopsis* leaf senescence (Gonzalez-Jorge et al., 2013). In Citrus fruits, however, the promotion of peel degreening and the reduction of carotenoids content induced by shading appears not to be related to the expression of *CCD4b1* (**Figure 9**) and either to the *CCD4a*, which is weakly expressed in fruit peel (Ma et al., 2013; Rodrigo et al., 2013b) and at similar low levels in covered and non-covered fruits (data not shown).

Similar to the expression of *CCD4b1*, analysis of transcripts accumulation related to key genes of the MEP pathway (*DXS*, *HDR1*, and *GGPPS1*) revealed a light-independent regulation (**Figure 8**). Moreover, this response was observed in both mandarins and oranges, indicating that the supply of precursors of the MEP pathway for carotenoids biosynthesis appears to be developmentally regulated during maturation and their expression levels are not a limiting factor for the reduction of carotenoids in shaded fruits. These results are in agreement with those obtained in red grapefruit (Lado et al., 2015b) and differ with the up-regulation of MEP genes by light during de-etiolation of *Arabidopsis* seedlings (Ruiz-Sola and Rodríguez-Concepción, 2012).

Analysis of the expression of seven genes of the carotenoid pathway in the peel of C and NC Clementine mandarin and Navelina orange revealed a marked stimulation of carotenoid biosynthesis by light (**Figures 6** and **7**). Results show that fruit shading down-regulated the transcription of all carotenoid biosynthetic genes in both citrus species, and this effect was more pronounced in upstream (*PSY*, *PDS*, and *ZDS1*) than in downstream genes (*LCY2a*, *LCY2b*, and *CHX*). Interestingly, differences in the expression between NC and C fruits were dependent of the gene and the species, but with the exception of *LCY2b*, stimulation of most genes during ripening were more noticeable in oranges than in mandarins, probably associated with the larger accumulation of carotenoids in the former. Previous studies in fruits of different Citrus species and varieties indicated that genes of the carotenoid biosynthetic pathway were up-regulated during ripening (Rodrigo et al., 2013a; Ikoma et al., 2016; Wei et al., 2017). Our results corroborate these observations and reveal a major stimulation of the expression of *PSY*, *ZDS*, *LCY2a*, *LCY2b*, and *CHX* genes in light-grown fruits of both mandarin and orange fruits. Other genes, as *PDS* and *LCY1* showed a variable expression in the peel of each variety. In contrast, fruit shading prevented but not abolished the induction of all the carotenoid biosynthetic genes analyzed. As earlier as in October, and before the major increment in gene expression, transcripts levels of the seven carotenoid biosynthetic genes studied were significantly lower in dark-grown mandarins than in those exposed to the light (**Figure 6**). In shaded Navelina oranges, the expression of *LCY2a*, *LCY2b*, and *CHX* gene were only downregulated at late maturity stages but upstream genes in the pathway were repressed from early maturation (**Figure 7**). It is worth to mention that even though in dark-grown fruits the expression of most carotenoid biosynthetic genes was arrested, a slow increment was detected as maturation progressed. Then, in mature fruits, accumulation of *PSY* was 2- and 3-times higher with respect to July fruits in mandarins and oranges, respectively, and the level of *LCY2b* increased 4-times in the peel of both varieties for the same period. This slow, but significant, stimulation of gene expression under darkness may explain the increment in carotenoid concentration observed in the peel of covered fruits at December (**Figures 3** and **5**). Collectively, these results indicate that light stimulates and sustains the expression of carotenoid biosynthetic genes in the peel of *Citrus* fruits and that under shading conditions a light-independent process may also operate. Down-regulation of carotenoid gene expression in response to shading was also reported in the red Star Ruby grapefruit, suggesting a conserved mechanism by which light stimulates transcription of carotenoid biosynthetic genes in fruits of different Citrus species (Lado et al., 2015b).

The light-driven upregulation of carotenoid biosynthetic genes has been largely documented in vegetative and reproductive tissues (Llorente et al., 2016b; Llorente et al., 2017). In Arabidopsis seedlings and tomato fruits, light stimulation of carotenoid metabolism is mediated by a complex molecular mechanism in which phytochrome interacting factors (PIFs) directly repressed the expression of *PSY1* gene (Toledo-Ortiz et al., 2010; Llorente et al., 2016a; Llorente et al., 2016b) and modulating the phase transition during ripening (Gupta et al., 2014). The differences observed in the expression of *PSY* and *LCYs* genes between light- and dark-grown oranges and mandarins are similar to that found in other systems (Fu et al., 2013; Llorente et al., 2016b). However, if the influence of light on the regulation of carotenoid biosynthesis in the peel of *Citrus* fruits is exerted by a direct mechanism similar to that operating in tomato fruits or other system, remains to be determined.

The important reduction in β,β-xanthophylls observed in the peel on covered mandarin and orange did not impair ABA content during the whole maturation process. Total carotenoid content in mandarin fruits in October, which were shaded two month before, was about 10% that of light-grown fruits (**Figure 4**) but the ABA content increase to a similar extent in both fruits (**Figure 10**). These results clearly indicated that the reduction of carotenoids by light deprivation is not sufficient to limit ABA synthesis in the peel and that the steps related to ABA formation are not substantially affected by shading. This response of mandarin and orange fruit to light avoidance is opposite to that found in red grapefruits, where bagging markedly impaired ABA accumulation (Lado et al., 2015b). This discrepancy may be related to the fact that lycopene accumulation in red grapefruit is the result of a blockage in conversion of lycopene to β-carotene, which may affect the availability of precursors for ABA synthesis (Lado et al., 2015b). In mandarin and oranges, it seems that even bagging produced a general shutdown of carotenoid synthesis, the xanthopllys pool is still enough to allow the formation of ABA.

In conclusion, shading of immature-green Clementine mandarin and Navelina orange fruits accelerated peel degreening and chlorophyll degradation, and markedly reduced total carotenoid content. Light avoidance does not substantially affect the expression of genes of the MEP pathway and *CCD4b1*, and the content of the apocarotenoid β-citraurin and ABA. Dark-grown fruits of both species experienced an increase in carotenoid content at later stages of maturity. It is then suggested that light stimulates carotenoid biosynthesis and accumulation in the peel of *Citrus* fruits but a light-independent regulation may also operate.

## Data Availability Statement

All datasets generated for this study are included in the manuscript/supplementary files.

## Author Contributions

JL and EA performed most of the experiments, carotenoid and gene expression analysis, data analysis, and wrote the first draft. MM and AG-C performed and analyzed ABA content. PC designed experiments and analyzed field experiments. MR and LZ conceived and designed the experiments, revised and wrote the final version of the manuscript. All the authors discussed the data and approved the final version of the manuscript.

## Funding

This work was supported by research grants AGL2012-34576 and AGL2015-70218 (Ministerio Economía y Competitividad, Spain). JL was recipient of a JAE-Predoc and EA a JAE-doc contract (CSIC-FSE). We acknowledge support of the publication fee by the CSIC Open Access Publication Support Initiative through its Unit of Information Resources for Research (URICI).

## Conflict of Interest

The authors declare that the research was conducted in the absence of any commercial or financial relationships that could be construed as a potential conflict of interest.
